# Parenchyma-Sparing Pancreatic Surgery: Current Indications, Results, and Future Prospects

**DOI:** 10.3390/cancers18101550

**Published:** 2026-05-11

**Authors:** Silvio Caringi, Antonella Delvecchio, Annachiara Casella, Valentina Ferraro, Matteo Stasi, Nunzio Tralli, Tommaso Maria Manzia, Michele Tedeschi, Riccardo Memeo

**Affiliations:** 1 Unit of Hepato-Biliary and Pancreatic Surgery, “F. Miulli” General Hospital, 70021 Acquaviva delle Fonti, Italy; 2 Department of Medicine and Surgery, LUM University, 70010 Casamassima, Italy; 3 Transplant and HPB Unit, Department of Surgery Sciences, University of Rome Tor Vergata, 00133 Rome, Italy

**Keywords:** pancreatic cancer, pancreatic surgery, parenchyma-sparing surgery

## Abstract

Parenchyma-sparing pancreatic surgery (PSPS) is a patient-centered alternative to traditional radical resections for benign and low-grade pancreatic lesions. Unlike pancreaticoduodenectomy and distal pancreatectomy, which tend to cause long-term exocrine and endocrine deficiency, PSPS aims to preserve functional tissue with a guarantee of oncologic safety. Techniques such as enucleation, central pancreatectomy, duodenum-preserving head resection, and uncinectomy are illustrative of this equipoise, with less risk of new-onset diabetes and malabsorption but more short-term morbidity in the form of postoperative pancreatic fistula. Advances in imaging technology, minimally invasive procedures, and robotics technology have extended PSPS indications beyond conventional candidates to thoughtfully selected neuroendocrine tumors, cystic neoplasms, and solid pseudopapillary neoplasms. Results are strongly dependent on patient selection, surgeon experience, and institutional volume, highlighting the importance of centralization and subspecialist training. While oncologic proficiency remains essential in aggressive tumors, evidence is in favor of PSPS being a curative and function-preserving option for properly screened patients with low-grade or benign conditions. Priorities for the future include multicenter prospective trials, optimization of perioperative techniques, and inclusion of patient-reported outcomes. PSPS represents a paradigm shift in pancreatic surgery, where technical innovation is balanced with quality of life in the long term and evolving principles of modern, individualized surgical practice.

## Simple Summary

Parenchyma-sparing pancreatic surgery (PSPS) stands out as an emerging and patient-focused approach to traditional, aggressive surgeries in treating benign and low-grade malignancies of the pancreas. Enucleations, central pancreatectomies, and duodenum-preserving operations are among the methods used to preserve endocrine and exocrine functions and ensure oncological safety in properly chosen patients. In comparison to traditional surgery, PSPS demonstrates better long-term results, minimizing the risk of diabetes and malabsorption syndrome; however, it is characterized by a higher rate of postoperative pancreatic fistula. Development in terms of imaging technologies, minimally invasive techniques, and robotics has enabled wider usage of PSPS, primarily in treating neuroendocrine and cystic tumors. The success of the procedure depends heavily on the accurate selection of patients, high surgical skills, and high caseload of an institution. Despite positive outcomes, existing evidence base is retrospective, underscoring the need for prospective multicenter trials.

## 1. Introduction

Pancreatic surgery has traditionally focused on major resections—pancreaticoduodenectomy (PD) for head tumors and distal pancreatectomy (DP) for body/tail disease—with an emphasis on oncologic resection for malignant tumors. While these remain the standards for invasive pancreatic adenocarcinoma and other high-grade malignancies, they unavoidably entail the removal of considerable amounts of exocrine and endocrine parenchyma and are followed by substantial short- and long-term morbidity, including pancreatic exocrine insufficiency, new-onset diabetes mellitus, and nutritional sequelae. Therefore, pancreatic tissue conservation is a main approach in the context of low oncologic risk and has encouraged the design and increasingly frequent application of parenchyma-sparing procedures such as enucleation (EN), central (or middle) pancreatectomy (CP), duodenum-preserving pancreatic head resection (DPPHR), and other limited resections for benign or low-grade neoplasms. This narrative review summarizes contemporary evidence on parenchyma-sparing pancreatic surgery, with particular emphasis on evolving indications for such procedures in the age of advanced imaging, enhanced perioperative care, and minimally invasive techniques [1,2].

## 2. Scope and Methods of the Review

The non-systematic review of the literature was conducted following the Preferred Reporting Items for Systematic Reviews and Meta-Analyses (PRISMA 2020) statement, as adapted for the environment of a non-systematic synthesis.

**Figure 1:**
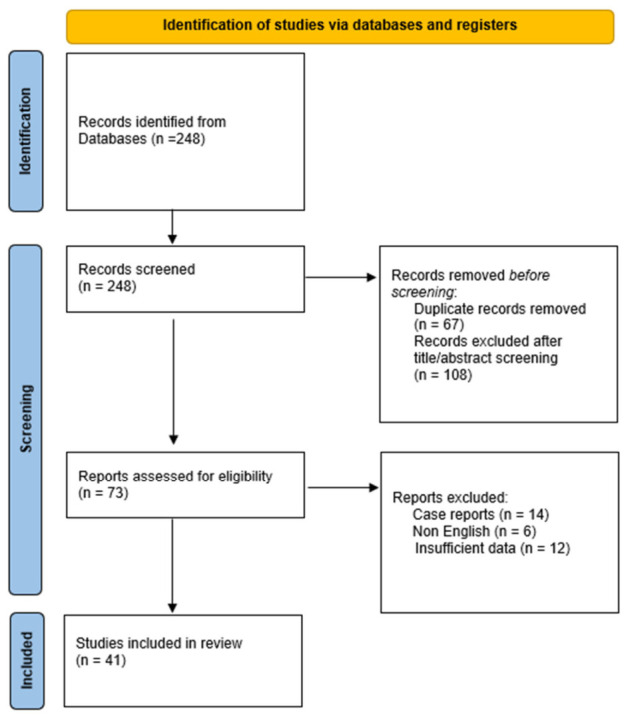
PRISMA-lite flowchart showing the selection of studies included in this descriptive review.

Figure 1 shows a PRISMA-lite flowchart illustrating the selection of studies included in this descriptive review.

The objective was to synthesize the existing evidence, indications, surgical techniques, and outcomes of parenchyma-sparing pancreatic surgery (PSPS).

A systematic literature review was carried out in the PubMed/MEDLINE, Scopus, and Web of Science databases for articles published between January 2010 and August 2025. The following keywords and Medical Subject Heading (MeSH) terms were combined using Boolean operators (“AND,” “OR”): “*parenchyma-sparing pancreatectomy*”, “*pancreatic enucleation*,” “*central pancreatectomy*,” “*duodenum-preserving pancreatic head resection*,” “*uncinectomy*,” “*pancreatic function preservation*,” and “*robotic pancreatic surgery*.”

The literature review was restricted to English-language articles on human beings. Duplicates were eliminated, and titles and abstracts were independently screened for eligibility according to pre-specified criteria.

The following inclusion criteria were applied:

1. Papers addressing indications, techniques, and perioperative or long-term outcomes of PSPS;

2. Systematic reviews, meta-analyses, large retrospective or prospective series, and expert consensus documents;

3. Full-text articles in English.

Exclusion criteria included case reports, editorials, animal studies, and conference abstracts without original data.

Discrepancies during selection were resolved by consensus with a senior reviewer. Reference lists of included studies and major reviews were hand-searched for additional eligible articles.

Data from the eligible studies were extracted into a standard template outlining study design, patient demographics, surgical technique, intra- and postoperative outcomes, and long-term functional outcomes.

Due to heterogeneity in patient groups, outcome definitions, and follow-up duration, no quantitative meta-analysis could be done. Thus, a qualitative synthesis was conducted in five important areas:

1. Evolution of indications for PSPS;

2. Technical aspects and surgical considerations;

3. Perioperative outcome and pancreatic fistula;

4. Long-term endocrine and exocrine function outcomes;

5. The role of minimally invasive and robotic techniques.

The methodological quality of systematic reviews and meta-analyses was assessed using the AMSTAR 2 checklist where applicable. The review was conducted according to the SANRA (Scale for the Assessment of Narrative Review Articles) guidelines to ensure clarity, rigor, and balanced reporting.

## 3. Rationale for Parenchyma-Sparing Surgery

The pancreas is blessed with a unique combination of endocrine and exocrine function; the destruction of large volumes of tissue can lead to clinically relevant diabetes and malabsorption. Parenchyma preservation aims to reduce these metabolic long-term sequelae while maintaining acceptable perioperative risk and oncologic safety where pathology allows. Indications were historically centered on small, benign tumors (e.g., insulinomas, serous cystadenomas) and low-grade neoplasms (certain pancreatic neuroendocrine tumors [pNETs], branch-duct IPMNs, and solid pseudopapillary neoplasms) but are being broadened as imaging and preoperative diagnostics improve risk stratification and minimally invasive and robotic techniques reduce operative morbidity. The desired balance involves optimizing functional preservation without compromising oncologic outcomes or tolerating excessive rates of procedure-specific complications such as postoperative pancreatic fistula (POPF) [1,3,4,5,6].

## 4. Technical Spectrum of Parenchyma-Sparing Procedures

Parenchyma-sparing pancreatic surgery represents one of the most fascinating evolutions in the field of pancreatic surgery. Its essence lies in the attempt to reconcile two imperatives that have traditionally been difficult to balance: on the one hand, the need to remove pancreatic lesions with adequate oncologic or functional safety and, on the other hand, the desire to preserve as much pancreatic tissue as possible, thereby maintaining long-term endocrine and exocrine function. Over the last three decades, a wide spectrum of techniques has been described and refined to meet these goals. Among them, pancreatic enucleation and central pancreatectomy are the most widely studied and adopted, but several variants—including duodenum-preserving head resections, uncinectomy, and individualized procedures for hereditary or multifocal disease—have also entered the surgical armamentarium. Understanding the technical spectrum of these approaches is critical for appreciating their indications, their limitations, and the potential advantages they may offer in selected patients.

### 4.1. Pancreatic Enucleation

Enucleation (EN) is the most primitive form of parenchyma-sparing resection, at least volumetrically, but it remains technically demanding. The technique of EN is simple: resect the lesion alone along its pseudocapsule or natural plane without sacrificing the overlying pancreatic parenchyma. However, the functional usage of this technique is not so straightforward. The lesion must be identified, mobilized carefully, and separated from the parenchyma without injury to the MPD or nearby vessels. For this reason, this surgery is traditionally reserved for small, benign, or low-grade tumors such as insulinomas, non-functioning neuroendocrine tumors smaller than two centimeters in diameter, and a few cystic neoplasms [7,8].

Preoperative work-up is essential. Cross-sectional, high-resolution imaging aided by magnetic resonance cholangiopancreatography (MRCP) allows for delineation of the ductal anatomy, as well as the proximity of the lesion to the MPD. Endoscopic ultrasound is commonly employed for diagnosis, as well as to assess the safety margin between the lesion and the duct; according to many authors [9], a distance of two to three millimeters or more should be ensured to prevent damage to the ducts. In addition to histological confirmation, we can also assess proliferation markers such as Ki-67, which is particularly relevant in pancreatic neuroendocrine tumors. Intraoperatively, the use of ultrasound is almost universal—not just to localize the lesion but also to guide the plane of dissection. It has been shown to reduce operative times and avoid unnecessary parenchymal sacrifice and inadvertent harm to the ducts [10].

Following lesion localization, dissection then generally proceeds with fine energy devices or bipolar cautery, but some surgeons still use cold dissection with a scalpel and scissors to prevent thermal spread. Traction on the lesion in a gentle manner will provide a natural plane to develop. When the MPD is near, dissection should be performed with particular caution. In suspected ductal injury, an attempt at direct repair can be made with fine absorbable sutures, or a drain can be placed to control the leak. The use of sealants has been attempted, but evidence of their efficacy has been mixed [11].

Minimally invasive approaches to EN have expanded dramatically. Laparoscopic EN has been practiced for more than two decades, with numerous studies showing comparable safety and faster recovery compared to open procedures [12]. More recently, robotic EN has emerged and been found to be particularly useful for lesions located in technically challenging regions such as the uncinate process or posterior head of the pancreas. The greater dexterity, steady visual axis, and three-dimensional visualization offered by robotic systems enable precise dissection of delicate structures. Indeed, recent series have documented reduced blood loss and reduced rates of conversion to open surgery with robotic EN compared with laparoscopic methods, although the danger of POPF remains, notably with lesions near the duct [13].

Nonetheless, EN has its disadvantages. The risk of POPF remains high—up to and sometimes greater than 30% in certain series—but the majority of fistulas are low-grade and can be managed conservatively [14]. For larger lesions more than three centimeters in diameter, those extending into the MPD, and those with presumed malignant potential, EN is generally not offered, as such patients are at higher risk of incomplete resection or complications. But in highly selected patients, EN is the optimal way to preserve pancreatic tissue and, hence, is a priceless operation in the parenchyma-sparing spectrum.

### 4.2. Central Pancreatectomy

If EN is the most limited resection, the most stereotypical parenchyma-sparing operation is central pancreatectomy (CP). This operation is conceptually beautiful: excise the diseased central segment of the pancreas—usually the neck and proximal body—while preserving the head and tail. This maintains a majority of the endocrine and exocrine parenchyma, avoiding the long-term metabolic changes of distal pancreatectomy. Nonetheless, the technical challenge of CP is considerable because the surgeon has to work with two transection surfaces of the pancreas and accomplish a successful reconstruction [15].

The indications for CP are narrowly defined. Benign or low-grade malignant lesions of the neck or proximal body of the pancreas, such as small neuroendocrine tumors, solid pseudopapillary tumors, or cystic lesions not involving the MPD, are good candidates. The procedure is contraindicated in pancreatic ductal adenocarcinoma, where broader margins and radical lymphadenectomy are required [16]. Preoperative imaging reiterates its significance, as precise representation of the lesion and ductal anatomy determines feasibility.

The technical aspects of CP begin with mobilization of the pancreas and careful identification of vascular landmarks—most importantly, superior mesenteric vessels and the portal vein. Proximal and distal transection of the pancreas is performed to remove the central segment. Management of the two stumps is the technical crux. The distal stump is usually left in continuity with the spleen and is permitted to drain into the gastrointestinal tract via its native relationships. The proximal stump must be reconstructed so that pancreatic secretions do not leak. Two techniques are popular: pancreaticojejunostomy (PJ) and pancreaticogastrostomy (PG) [17].

PJ is the more traditional approach and is most frequently performed as a Roux-en-Y anastomosis. Pancreatic secretions drain into a defunctionalized loop of the jejunum. The technique can be performed by a duct-to-mucosa suture when the duct is visible or by an invagination technique when the duct is very small. PG, however, directly approximates the pancreatic stump to the posterior wall of the stomach. Its proponents argue that this provides a good vascular recipient site, a shorter operative distance, and possibly a lower risk of anastomotic disruption due to the acidity of the gastric environment. Comparative data between PG and PJ are limited, and outcomes appear virtually indistinguishable; the choice is typically dependent upon surgeon preference and regional familiarity [18].

Minimally invasive CP, whether laparoscopic or robotic, is highly demanding due to the need for fine intracorporeal suturing. Robotic technology has made this transition feasible, with added dexterity and precision. Early CP series of robots have shown feasibility and acceptable complication rates, though the complication rate of POPF remains higher than that of standard distal pancreatectomy [19]. The continued risk is the price for maximal parenchymal preservation. Long-term outcomes always show superior preservation of endocrine and exocrine function following CP compared to distal pancreatectomy, making its application in appropriately selected patients worthwhile [20].

### 4.3. Duodenum-Preserving Head Resections and Other Limited Techniques

While EN and CP are the most frequently reported parenchyma-sparing procedures, there is a broader technical spectrum. Lesions confined to the uncinate process or limited to the partial head of the pancreas can sometimes be managed with procedures not involving a Whipple resection while also sparing the duodenum. Uncinectomy is an example of an operation where only the uncinate process is resected, while the remaining head is preserved. This involves meticulous dissection across the superior mesenteric vessels and pancreaticoduodenal arcades while managing to avoid the bulky reconstruction required after pancreaticoduodenectomy [21].

Duodenum-preserving pancreatic head resection (DPPHR), initially designed for chronic pancreatitis, has also been applied to selected benign and low-grade tumors of the head. The procedure involves excision of diseased head tissue without duodenal sacrifice or bile-duct and gastric outflow. Technically, it is a balancing act here: enough tissue must be removed to excise the lesion or inflamed parenchyma, but the blood supply of the duodenum and biliary tree must be carefully preserved. Reconstruction is typically done by anastomosis of the jejunum to the pancreatic remnant. Modifications such as the Beger, Frey, and Berne procedures illustrate how different philosophies in surgery have addressed this technical challenge [22].

Robotic technology has, again, joined in encouraging these less common procedures. Several reports document robotic uncinectomy or partial head resections with acceptable morbidity, capitalizing on enhanced robotics to navigate small anatomic spaces. While experience currently is only available in high-volume centers, these advances expand the possibility of performing parenchyma-sparing resections in challenging anatomic locations [23].

Although distinct in terms of details, parenchyma-sparing procedures all share identical technical concerns. One of these is the risk of POPF, which is always higher after EN and CP compared to postoperative risk following conventional pancreaticoduodenectomy or distal pancreatectomy. This is because the transection surfaces is minimal, the pancreatic tissue is soft, and the duct is typically not dilated, all of which are risk factors for leakage [24]. The placement of drains near the site of resection continues to be routine in most centers, although the optimum method for drain management is still debated. Preventive stenting of the MPD in well-chosen EN cases, particularly if the lesion lies at the tip of the duct, is favored by some surgeons, even though there is conflicting data on its utility [25].

A further chronic issue is the learning curve. These operations demand high skill in pancreatic dissection, intracorporeal suturing, and complication management. Even for high-volume institutions, CP and complex EN are performed relatively rarely, providing surgeons with limited practice to become proficient. Thus, outcomes are significantly associated with institutional and surgeon volume [26]. This reality underpins the requirement for centralization of services and for intensive training programs to disseminate expertise.

Table 1 shows a comparative summary of parenchyma-sparing techniques, summarizing indications, technical aspects, advantages, and disadvantages.

## 5. Indications: Traditional and Evolving

The role of indications for parenchyma-sparing pancreatic resections has significantly changed during the past three decades. Historically, pancreatic surgery was ruled by the principle of radicality: operations were planned and carried out with the primary concern of oncologic safety and avoidance of postoperative complications, sacrificing the function of the pancreas. Operations such as pancreaticoduodenectomy and distal pancreatectomy, though very successful in managing malignant disease, were used indiscriminately, even on benign or indolent tumors. The result was a high morbidity of long-term exocrine and endocrine deficiency, with diabetes and malabsorption severely debilitating the life of patients who, in the majority of cases, would have otherwise enjoyed virtually normal survival [27,28].

Against this background, parenchyma-sparing surgical methods such as enucleation (EN), central pancreatectomy (CP), duodenum-preserving head resection (DPPHR), and uncinectomy were initially conceived as super-special optionals, primarily for benign or borderline lesions in which radical resection was not deemed to be indicated. Over time, improvements in imaging, perioperative care, and operating room technology—most importantly, the advent of minimally invasive and robotic modalities—have facilitated a paradigm shift. Increasingly, these techniques are viewed less as compromises and more as strategic, carefully considered maneuvers aimed at maximizing long-term functional preservation with a minimal loss of safety. To understand this evolution, it is useful to define the traditional indications and the new, more extensive criteria that have developed.

### 5.1. Traditional Indications

PSPS is primarily indicated for selected benign and low-grade malignant lesions, including solid pseudopapillary neoplasms, mucinous cystic neoplasms, and pancreatic neuroendocrine tumors (such as insulinomas and gastrinomas). These lesions are frequently diagnosed incidentally during imaging performed for unrelated conditions, whereas symptomatic presentation is often nonspecific and may delay accurate diagnosis.

#### 5.1.1. Benign Neuroendocrine Tumors

Parenchyma-sparing surgery was once best known for the management of benign or low-grade neuroendocrine tumors (NETs)—most notably, insulinomas. These tiny, frequently solitary tumors are most frequently located in the tail or body of the pancreas and virtually never metastasize. Decades ago, enucleation was considered the gold standard for insulinomas due to their benign nature and well-defined capsule, facilitating secure removal [12]. Central pancreatectomy and, less frequently, uncinectomy or limited head resection were carried out in lesions inappropriate for straightforward enucleation due to anatomical considerations.

With the enhancement of preoperative localization techniques—most notably with endoscopic ultrasonography (EUS), somatostatin receptor scintigraphy, and intraoperative ultrasonography—the safety margin of EN has widened. It has been demonstrated in various studies that in the event of small functioning NETs—most notably, insulinomas EN provides superb long-term outcomes, with resection cure rates comparable to conventional resections and with the benefit of preserved pancreatic function [29].

#### 5.1.2. Non-Functioning Neuroendocrine Tumors

Non-functioning NETs have previously been treated more aggressively due to their higher risk of malignant potential compared to functioning NETs. However, lesions that are smaller than 2 cm, well differentiated on biopsy, and without evidence of radiological invasion or metastases have always been perceived to be amenable to EN or CP [7]. In these cases, the intention is to prevent overtreatment: excision of a lesion, potentially with little impact on survival, with the prevention of morbidity of resection in great abundance.

#### 5.1.3. Cystic Neoplasms

A second classic indication is represented by selected cystic neoplasms. Serous cystadenomas, for instance, are almost always benign and need to be operated on only if symptomatic or sufficiently large to cause compressive symptoms. In these cases, parenchyma-sparing resection was often employed to avoid unnecessary loss of normal tissue [30]. Similarly, MCNs with low-grade dysplasia in the neck or body of the pancreas have been treated by CP, where distal pancreatectomy would create a significant functional deficit. With IPMNs, on the other hand, the threshold was traditionally higher, since the risk of malignancy usually determined standard resection.

#### 5.1.4. Solid Pseudopapillary Neoplasms

Solid pseudopapillary neoplasms (SPNs), despite their malignancy potential, usually present in young females and are also linked to an indolent course and favorable prognosis after complete resection. For tumors of the neck or proximal body, CP has long been a legitimate alternative, yielding oncologic clearance with preservation of the pancreatic reserve [31].

In all of these traditional indications, the unifying principle is that parenchyma-sparing techniques were reserved for benign or low-grade malignant lesions without a sign of ductal adenocarcinoma or invasive pathology. The rationale was pragmatic: save tissue when radicality is not necessary.

### 5.2. Expanding Indications: The Evolving Landscape

#### 5.2.1. Broader Criteria for Neuroendocrine Tumors

The most significant improvement has been in the management of non-functioning NETs. Current recommendations, based on large cohorts and registries, increasingly prefer a selective parenchyma-sparing approach, even for lesions of up to 3 cm in diameter, provided they are well differentiated and not associated with nodal or distant metastases [32]. EN and CP are no longer procedures of last resort but are actually part of the management protocol of small, localized NETs.

Furthermore, the treatment of multifocal NETs, particularly in hereditary syndromes such as MEN1, has facilitated increased application of parenchyma-sparing surgery. In MEN1, there are many small NETs present, leading to the question of whether large resection or multiple enucleations and limited resections should be undertaken. The second approach, although technically demanding, is in keeping with the general philosophy of preserving as much native pancreatic function as is possible in those who have recurrent and frequent need of repeated interventions over their lifespan [33].

#### 5.2.2. Cystic Neoplasms in the Era of Refined Imaging

New indications are most evident in cystic neoplasms. Advances in imaging and cyst fluid analysis have made it more potent to stratify risk and distinguish lesions that can be resected from those that can be watched. Where operation is required, parenchyma-sparing resections are increasingly favored for non-high-risk lesions. For instance, branch-duct IPMNs with no mural nodules and no main duct extension can be treated by EN or CP if they are located in ideal positions [34]. This is within a larger trend of personalization of surgical decision-making between malignancy risk and the cost of functional loss.

#### 5.2.3. Solid Pseudopapillary Neoplasms Reconsidered

The role of parenchyma-sparing resection in SPNs has also expanded. While formal resections remain the gold standard for large or aggressive lesions, small, well capsulated SPNs are increasingly managed by CP or even EN in highly selected cases [35]. The fact that complete capsule-preserving resection is adequate to ensure oncologic control has made it reasonable to apply a more conservative surgical strategy. Particularly in younger patients, long-term pancreatic reserve preservation is precious, given the excellent prognosis of these tumors.

#### 5.2.4. Pancreatic Neuroendocrine Tumors and Functional Preservation

The growing interest in postoperative quality of life has refocused interest in the preservation of function, even in oncologic contexts in which radicality was never questioned in the past. Current prospective evidence alludes to the high prevalence of new-onset diabetes and exocrine insufficiency after distal pancreatectomy or pancreaticoduodenectomy, especially in young patients [36]. Against this background evidence, the plea for parenchyma-sparing surgery has become even more potent, going beyond the strict confines of benign disease.

One of the main drivers of indication expansion has been progress in minimally invasive and robot-assisted surgery. Early concerns regarding the technical viability of complex parenchyma-sparing procedures via laparoscopic or robotic platforms have largely been addressed. In robotic surgery, in fact, procedures such as CP, DPPHR, and uncinectomy are rendered more replicable, with improved dexterity and accuracy, allowing for safe dissection near delicate structures [13,23].

As morbidity from surgery falls, the cut-off for offering parenchyma-sparing resections has fallen. Surgeons are now more at ease in offering EN for more obtuse lesions, CP for challenging central tumors, and even DPPHR for chosen head lesions for which pancreaticoduodenectomy would previously have been the first choice [16]. Thus, technology has acted not only as an enabler but also as a change catalyst.

Perhaps the most important determinant of changing indications is the growing appreciation of functional results. All long-term series consistently demonstrate that EN and CP are superior to traditional resection in preserving both exocrine and endocrine function [26]. Perhaps most importantly, new-onset diabetes following CP is considerably less than following distal pancreatectomy, demonstrating the advantage of tissue preservation. Conversely, EN has minimal risk for exocrine deficiency but a higher risk for POPF. These results justify the push to expand indications: if oncologic security is not compromised, preservation of function is the primary goal.

This focus is particularly noteworthy in young patients, who face decades of life with the metabolic consequences of pancreatic insufficiency. In young patients, the tolerance for offering parenchyma-sparing surgery has become more liberal, even for lesions previously treated with radical resection [24,36,37].

## 6. Perioperative Outcomes and Trade-Offs

### 6.1. Postoperative Pancreatic Fistula (POPF)

POPF remains the signature complication of parenchyma-sparing operations, especially EN and CP. Rates of clinically significant (Grade B/C) POPF are highly variable between series, but CP, in particular, has been associated with high POPF rates (systematic reviews quote clinically significant POPF at 20–30% in some collections), contributing to global morbidity and extended hospital stays. EN is also associated with increased fistula rates compared with routine resections in some cohorts, but fistulas following EN usually have a distinctive clinical course and can be treated conservatively in most instances. Methods of preventing POPF—meticulous transection of the gland, use of biological adhesives, strict drain management, and judicious use of somatostatin analogs—are inconsistently practiced, and evidence supporting prophylactic maneuvers is still heterogeneous. The preference for parenchymal preservation will therefore weigh the risk of morbidity of POPF against long-term metabolic benefits [4,38].

### 6.2. Endocrine and Exocrine Function

Pancreatic tissue preservation generally translates to improved late endocrine outcomes. Meta-analyses have shown decreased incidences of new-onset diabetes after CP in comparison with distal pancreatectomy and better endocrine outcomes after EN in comparison with standard resections because of the lower islet-cell mass loss. Exocrine function is also more prolonged but less well documented to be reliable. Functional benefits are likely the major long-term justification for parenchyma-sparing therapies, particularly in youth and patients with hereditary or multifocal disease. However, unwarranted perioperative morbidity can negate gains in the short term, and long-term quality-of-life data remain sparse [1,5].

### 6.3. Oncologic Safety

Oncologic outcomes are lesion-dependent. For benign and borderline tumors, parenchyma-sparing resections are curative in the vast majority of cases when patient selection is appropriate. For low-grade malignancies (well-differentiated pNETs and SPNs), retrospective series suggest comparable disease-specific survival for selected patients treated with EN/CP versus formal resections, but long-term data are heterogeneous, and selection bias is a concern in observational series. For lesions with higher malignant potential or suspicious imaging/biopsy characteristics, standard oncologic resections with lymphadenectomy remain indicated. Vigilant long-term surveillance is recommended after any limited resection performed for borderline or low-grade malignant disease [39,40].

### 6.4. Minimally Invasive and Robotic Approaches

Minimally invasive (laparoscopic and robotic) techniques have found applications in EN, CP, and DPPHR with increasing frequency. Robotic platforms, in particular, provide added dexterity and suturing capacity, which facilitate parenchyma-sparing reconstructions such as pancreaticojejunostomy and fine enucleation near the duct. Systematic reviews of robotic parenchyma-sparing pancreatectomy show feasibility and safety in high-volume centers, with potential advantages in operative blood loss, length of stay, and conversion rates compared to laparoscopy. However, evidence is primarily derived from single-arm series or cohort comparison trials; there are no good-quality comparative trials, and recommendations remain at a low evidence level. Cost, the learning curve, and the volume of cases are important for centers that are thinking of implementing such interventions [41,42].

## 7. Strategies to Reduce Complications and Optimize Outcomes

Perioperative and intraoperative strategies aimed at reducing POPF and related morbidity after parenchyma-sparing surgery are summarized in Table 2.

## 8. Discussion

### 8.1. Rationale and Indications

The available evidence on PSPS is characterized by substantial heterogeneity. Divergences in tumor biology (neuroendocrine tumors, cystic lesions, and solid pseudopapillary tumors), surgical approach (enucleation, central pancreatectomy, and duodenopancreatectomy), research methodology, and outcome definition pose serious obstacles to comparative analysis. Accordingly, outcomes should be assessed relative to the clinical situation rather than universally applied to PSPS as a whole.

The findings of this narrative review emphasize the revolution in philosophy and approach to pancreatic surgery that has been achieved over the past three decades. Historically, the specialty has been characterized by a radical resection dogma, epitomized by PD and DP, procedures that support oncologic clearance, even in the context of benign or borderline lesions. The outcome of such a philosophy was one generation of endocrine and exocrine sequelae-compromised patients with new-onset diabetes mellitus, malabsorption, steatorrhea, and the long-term burden of nutritional deficiency. The evolution of PSPS is a deliberate attempt to rebalance this trade-off: to avoid unnecessary sacrifice of functional parenchyma without compromising safety, particularly in the setting of benign or low-grade lesions.

The essential contribution of PSPS is conceptual rather than technical: it is a shift in surgical ethics from disease-oriented to patient-oriented philosophy. The implicit realization is that in most benign or low-grade pancreatic neoplasms, quality of life over the long term, as defined by pancreatic function, is no less a consideration than local control of disease. This transformation in priorities has been enabled through technological advances, enhanced risk stratification through imaging and histology, and the evolution of minimally invasive and robotic platforms. In this new paradigm, parenchyma-sparing techniques have emerged not as bizarre exceptions but as a usual option in well-screened populations of patients.

The primary rationale for PSPS is superior preservation of endocrine and exocrine function. CP, per se, in contrast to distal pancreatectomy, markedly reduces the risk of postoperative diabetes due to the preservation of both the head and tail of the gland. EN, by resecting only the tumor and leaving almost all of the parenchyma intact, best preserves function, particularly in young patients with several years of life expectancy. These advantages are considerable: pancreatic exocrine deficiency and diabetes mellitus are chronic conditions resulting in serious compromise of quality of life, decreasing nutritional status, and increasing cardiovascular morbidity.

### 8.2. Balance Between Benefits and Risks

Against the certain functional benefit stands the ongoing risk of POPF, the “Achilles’ heel” of PSPS. POPF rates are always higher after EN and CP than after PD or DP, primarily due to the soft nature of the saved pancreatic tissue, the minute caliber of ducts, and the technical challenge involved in operating in more than one plane of transection. While most are low-grade and can be treated conservatively, clinically relevant fistulas prolong hospital stays; increase readmission; and, in some cases, lead to life-threatening complications. The PSPS paradox is therefore a delicate balance: better long-term outcomes are offered by procedures with higher morbidity in the short term. Counterbalancing this paradox requires patient choice, technical ability, and institutional expertise. Relative to PD and DP, PSPS places the cost of radicality into focus. PD remains unmatched for head ductal adenocarcinoma when oncologic integrity is paramount, but DP remains the gold standard for body and tail malignant or invasive lesions. However, in benign and borderline cases, these procedures have functional costs that become increasingly difficult to justify. A large series of patients treated with DP for benign tumors reports a diabetes incidence of close to 40%, which is considerably more than the negligible oncologic benefit of such radical resections in low-grade tumors. In contrast, studies on CP report significantly lower incidences of new-onset diabetes, and EN preserves function in the great majority of patients, with no real long-term metabolic burden.

This analogy illustrates the logic of modifying surgery to pathology: radical surgery for virulent disease and parenchyma-sparing maneuvers when oncologic risk is low. The analogy to organ-sparing surgery elsewhere—e.g., breast-conserving surgery in neoplasm or nephron-sparing therapy in urology—is instructive. Just as lumpectomy replaced radical mastectomy in well-screened patients, PSPS is taking the place of PD and DP in the domain of benign and low-grade pancreatic neoplasms.

### 8.3. Patient Selection and Current Limitations

PSPS depends heavily on judicious patient selection. In defining ductal anatomy, vascular connections, and the relationship with the main pancreatic duct, multiphase CT, MRI, and MRCP are critical. Endoscopic ultrasonography, with the ability to characterize small lesions and fine-needle aspiration, contributes additional accuracy to decision-making. Small, well-differentiated neuroendocrine tumors, cystic tumors with no concerning features, and encapsulated solid pseudopapillary tumors are straightforward candidates. In hereditary conditions such as MEN1, where multifocality is common, the role of several enucleations or staged, limited resections merely serves to emphasize, again, the priority of individualization.

The patient’s metabolic state and age are also key determinants. Younger patients derive the most advantage from preservation of pancreatic function, as new-onset diabetes at younger ages provides a longer burden of disease and heightened lifetime risk of complications. Conversely, in older patients and those with limited remaining life expectancy, the panoply between preserving parenchyma and endangering POPF can lean toward more traditional resections. This math demonstrates that PSPS is not an ideological commitment to parenchymal protection at all costs but a rational strategy to optimize outcomes on an individual basis.

Particular attention should be paid to the use of somatostatin analogs, which, although commonly employed in the management of pancreatic neuroendocrine tumors, may induce gallbladder hypomotility and increase the risk of gallstone formation. This potential complication should be considered, as it may occasionally require additional surgical intervention [43].

Institutional volume and surgeon experience are very sensitive to the implementation of PSPS. EN and CP are technically demanding procedures that require precise intraoperative ultrasound guidance, cautious dissection around friable ductal and vascular elements, and advanced reconstructive maneuvers. The rarity of suitable cases also contributes to the challenge: even at high-volume institutions, CP is a relatively low-frequency procedure. Outcomes are therefore closely related to institutional volume. High-volume institutions all have lower morbidity and mortality, while outcomes at low-volume institutions are not so good. The volume–outcome relationship has led proponents of centralization of PSPS to specialized institutions following the path of complicated oncologic pancreatic surgery.

### 8.4. Future Directions

The contribution of robotic technology to spanning of the learning curve warrants special mention. Robotic systems offer greater dexterity, stable three-dimensional imaging, and better ergonomics, all combining to enable such delicate actions as intracorporeal suturing in CP and precise dissection in EN. Several series now indicate that robotic PSPS is associated with lower rates of conversion and better perioperative outcomes than laparoscopy, although randomized data are limited. However, robotics appear to be an enabling technology capable of enhancing the feasibility and safety of PSPS in anatomically challenging locations.

The remaining question is whether PSPS is oncologically sufficient in low-grade malignant tumors. In benign tumors, the issue is not total resection, with or without EN, CP, or DPPHR, but curing. However, for low-grade malignancies such as well-differentiated neuroendocrine tumors or solid pseudopapillary neoplasms, the evidence is less clear-cut. Retrospective series that are biased towards reporting similar disease-specific survival in PSPS and standard resections are marred by selection bias, as well as a lack of randomized trials. Furthermore, lymphadenectomy is not commonly achieved in PSPS, with attendant questions of occult nodal disease.

These restrictions mandate intensive postoperative surveillance. Patients treated with PSPS for low-grade malignancies need to be enrolled in systematic follow-up programs with the use of cross-sectional imaging; biochemical monitoring; and, if necessary, functional assessment. This enables prompt detection of recurrence and permits timely intervention. The emphasis on surveillance is part of a global philosophy: PSPS is less of a technical operation but, rather, one component of an expanded regimen of patient management that integrates surgical, medical, and radiologic expertise. It is vital to note that most studies are based on retrospectively analyzed single or multicenter case series, with few systematic reviews and meta-analyses and no randomized controlled trials. In other words, the quality of existing data is relatively low and ranges from low to moderate.

Some trends will shape the direction of PSPS in the future. Firstly, advances in intraoperative imaging—namely, real-time navigation with augmented reality and indocyanine green fluorescence—can potentially enhance lesion localization, delineate ductal anatomy, and reduce the risk of ductal injury. Secondly, predictive machine learning models and multicenter registries have the potential to risk-stratify patients for POPF and thereby enable individualized perioperative care. Third, there exists an imperative to include higher-level evidence of comparison between PSPS and traditional resections in both in functional and oncologic outcomes in future multicenter trials. In addition, preoperative 3D reconstruction is increasingly used to enhance surgical planning, particularly in complex anatomical settings. Emerging applications of artificial intelligence may further support surgical decision-making and intraoperative navigation in the near future.

In parallel, research into perioperative care has the potential to reduce morbidity. Tactical protocols for care of drains, pharmacoprophylaxis with somatostatin analogs, and refinement of anastomotic methods could dampen the burden of POPF. Robotic technology, if combined with a rigorous training curriculum, is capable of making technically demanding PSPS available to more than a handful of high-volume institutions.

Besides the technical aspects, there has been increased emphasis on quality-of-life issues and functional outcome considerations, which have become essential factors in surgical decision-making for pancreatic disease patients. This tracks with broader trends in surgical ethics and oncology, in which survivorship and patient-reported outcomes become increasingly focal to decision-making. PSPS is therefore not solely a technical innovation but part of a paradigm shift toward humanist decision-making and patient-centered care in pancreatic disease.

In summary, PSPS should no longer be viewed as a one-size-fits-all alternative to standard resections but as an individualized approach for carefully selected patients in high-volume, multidisciplinary centers. The development of international registries, the standardization of functional outcome definitions, and the incorporation of patient-reported quality-of-life metrics into clinical decision-making algorithms must be the way ahead.

## 9. Limitations

This review has several limitations. First, it is a narrative synthesis, and although conducted in accordance with PRISMA principles, it lacks the quantitative analysis framework of a full systematic review. Second, the included studies are heterogeneous in terms of design, patient selection, and outcome reporting, including inconsistent definitions of key endpoints such as postoperative pancreatic fistula (POPF), often variably defined according to different versions of the ISGPS criteria, and endocrine insufficiency, which limits direct comparison and meta-analytic interpretation. Third, most of the data are from retrospective series in high-volume centers, with potential for publication and selection bias. Moreover, long-term outcomes, particularly quality-of-life and patient-reported measures, remain under-reported and heterogeneous, limiting the strength of conclusions regarding functional superiority. Lastly, long-term functional and oncologic results following parenchyma-sparing pancreatic resections continue to be inconsistently reported, highlighting the necessity of prospective multicenter registries and the standardization of outcome definitions.

## 10. Conclusions

PSPS is increasingly considered an option complementary to standard resections, particularly for benign and low-grade pancreatic lesions. Its central value lies in preserving both endocrine and exocrine function, thereby potentially reducing the long-term burden of diabetes, malabsorption, and nutritional insufficiency that frequently follow pancreaticoduodenectomy or distal pancreatectomy. Enucleation, central pancreatectomy, and duodenum-preserving resections exemplify a patient-centered approach that balances oncologic adequacy with functional preservation. Indeed, PSPS may be associated with similar oncologic results in appropriately selected patients, but the current literature is primarily retrospective and prone to selection bias.

Despite these advantages, PSPS remains technically demanding and is associated with higher rates of postoperative pancreatic fistula compared with conventional resections. These complications, while often manageable, highlight the need for rigorous patient selection, meticulous surgical technique, and delivery within high-volume centers where institutional expertise can mitigate risks. Importantly, the absence of extensive lymphadenectomies limits the applicability of PSPS in high-grade malignancies, underscoring that radical resections remain essential for pancreatic adenocarcinoma and other aggressive neoplasms.

The ongoing refinement of imaging modalities, minimally invasive techniques, and robotic platforms is likely to expand the safety and feasibility of PSPS. Future progress will depend on prospective multicenter studies that define long-term oncologic safety, establish standardized strategies to reduce morbidity, and integrate patient-reported outcomes into surgical decision-making.

In summary, PSPS represents a paradigm shift from a purely radical philosophy toward individualized, function-preserving treatment. However, these approaches should currently be reserved for carefully selected patients in high-volume centers, given the lack of high-level evidence supporting broader adoption. In these cases, PSPS may lead to tumor control without compromising long-term quality of life, but well-designed prospective studies are still lacking.

## Figures and Tables

**Table 1:** Comparative table of parenchyma-sparing techniques.

Procedure	Typical Indications	Technical Highlights	Advantages	Limitations/Risks	Long-Term Outcomes
Enucleation (EN)	Small benign or low-grade lesions	-Local dissection along the pseudocapsule -Intraoperative ultrasound is critical. -Duct proximity <2–3 mm increases risk	-Maximal parenchymal preservation -Avoids reconstruction. -Short operative time	-High rate of POPF (20–40%) -It is not indicated if the lesion has malignant potential. -Risk of incomplete resection	-Excellent preservation of endocrine/exocrine function. -Recurrence is rare if appropriately selected
Central Pancreatectomy (CP)	Benign or low-grade lesions in the pancreatic neck/proximal body	-Resection of neck/body with preservation of head and tail. -Requires reconstruction (PJ or PG)	-Preserves more pancreatic tissue than distal pancreatectomy -Reduced risk of diabetes and steatorrhea	-Technically demanding; two transection surfaces. -Higher POPF rates than distal pancreatectomy. -Not suitable for PDAC	-Superior preservation of pancreatic function vs. distal pancreatectomy. -Durable outcomes in benign/low-grade tumors
Duodenum-Preserving Head Resection (DPPHR)	-Chronic pancreatitis with head-dominant disease. -Selected benign/low-grade tumors of the head	-Resection of pancreatic head tissue while preserving the duodenum and bile duct. -Delicate preservation of vascular arcades	-Avoids the morbidity of pancreaticoduodenectomy. -Preserves gastric and biliary continuity	-Technically complex; risk of duodenal ischemia or biliary stenosis. -Limited oncologic role	-Effective pain control in pancreatitis. -Good preservation of function in benign tumors
Uncinectomy	-Isolated lesions of the uncinate process	-Resection limited to the uncinate. -Dissection close to SMV, SMA, and pancreaticoduodenal vessels	-Avoids full pancreatic head resection. -Preserves the duodenum and the bile duct	-High technical difficulty. -Risk of vascular injury. -Not widely performed outside high-volume centers	-Functional preservation is successful. -Low recurrence for benign lesions

**Table 2:** Strategies to reduce complications.

Preoperative Optimization	Nutritional Assessment, Glycemic Control, and Careful Patient Selection Based on Gland Texture and Duct Size
Intraoperative Imaging	Routine use of intraoperative ultrasound to map tumor–duct relations and guide resection planes.
Technical Refinements	Parenchymal suturing techniques, selective duct ligation or stenting when warranted, and thoughtful reconstruction (e.g., pancreaticogastrostomy vs. pancreaticojejunostomy) for CP.
Drai Management	Selective and protocolized drain strategies informed by early drain-fluid amylase measurements.
Perioperative Pharmacology	Selective use of somatostatin analogs in high-risk patients remains controversial but is sometimes practiced.

## Data Availability

No new data were created or analyzed in this study. Data sharing is not applicable to this article.

## Abbreviations

**Abbreviation**	**Definition**
PSPS	Parenchyma-Sparing Pancreatic Surgery
EN	Enucleation
CP	Central Pancreatectomy
DPPHR	Duodenum-Preserving Pancreatic Head Resection
POPF	Postoperative Pancreatic Fistula
PD	Pancreaticoduodenectomy
DP	Distal Pancreatectomy
MPD	Main Pancreatic Duct
pNET	Pancreatic Neuroendocrine Tumor
SPN	Solid Pseudopapillary Neoplasm
MCN	Mucinous Cystic Neoplasm
IPMN	Intraductal Papillary Mucinous Neoplasm
MEN1	Multiple Endocrine Neoplasia type 1
MRCP	Magnetic Resonance Cholangiopancreatography
EUS	Endoscopic Ultrasound
PJ	Pancreaticojejunostomy
PG	Pancreaticogastrostomy
SANRA	Scale for the Assessment of Narrative Review Articles
PRISMA	Preferred Reporting Items for Systematic Reviews and Meta-Analyses
AMSTAR 2	A Measurement Tool to Assess Systematic Reviews, version 2
